# Eventually Periodic Solutions of a Max-Type Difference Equation

**DOI:** 10.1155/2014/219437

**Published:** 2014-07-01

**Authors:** Taixiang Sun, Jing Liu, Qiuli He, Xin-He Liu, Chunyan Tao

**Affiliations:** ^1^College of Mathematics and Information Science, Guangxi University, Nanning, Guangxi 530004, China; ^2^College of Electrical Engineering, Guangxi University, Nanning, Guangxi 530004, China

## Abstract

We study the following max-type difference equation *x*_*n*_ = max⁡{*A*_*n*_/*x*_*n*−*r*_, *x*_*n*−*k*_}, *n* = 1,2,…, where {*A*_*n*_}_*n*=1_^+*∞*^ is a periodic sequence with period *p* and *k*, *r* ∈ {1,2,…} with gcd(*k*, *r*) = 1 and *k* ≠ *r*, and the initial conditions *x*_1−*d*_, *x*_2−*d*_,…, *x*_0_ are real numbers with *d* = max⁡{*r*, *k*}. We show that if *p* = 1 (or *p* ≥ 2 and *k* is odd), then every well-defined solution of this equation is eventually periodic with period *k*, which generalizes the results of (Elsayed and Stevi$\overset{´}{\text{c}}$ (2009), Iričanin and Elsayed (2010), Qin et al. (2012), and Xiao and Shi (2013)) to the general case. Besides, we construct an example with *p* ≥ 2 and *k* being even which has a well-defined solution that is not eventually periodic.

## 1. Introduction

The max operator arises naturally in certain models in automatic control theory (see [1]). In recent years, the discrete case involving difference equations with maximum has been receiving increasing attention, for some results in this area; see, for example, [2–4].

In this paper, we consider the following max-type equation:

(1) $$\begin{array}{c} x_{n}=\underset{}{\max}\left\{\frac{A_{n}}{x_{n-r}},x_{n-k}\right\},\;n=1,2,\ldots , \end{array}$$

where {*A*_*n*_}_*n*=1_^+*∞*^ is a periodic sequence with period *p* and *k*, *r* ∈ {1,2,…} with gcd(*k*, *r*) = 1 and *k* ≠ *r*, and the initial conditions *x*_1−*d*_, *x*_2−*d*_,…, *x*_0_ are real numbers with $d=\underset{}{\max}\left\{r,k\right\}$.

In [5], Iri$\overset{ˇ}{\text{c}}$anin and Elsayed showed that every well-defined solution of (1) is eventually periodic with period 4 when *k* = 4, *r* = 1, and *p* = 1. Elsayed and Stevi$\overset{´}{\text{c}}$ [6] showed that every well-defined solution of (1) is eventually periodic with period 3 when *k* = 3, *r* = 1, and *p* = 1. In [7], Xiao and Shi showed that if *k* = 2, *r* = 1, and *p* = 1, then every well-defined solution of (1) is eventually periodic with period 2. Qin et al. [8] showed that every well-defined solution of (1) is eventually periodic with period *k* when *r* = 1 and *p* = 1.

In this paper, we will generalize the results of [5–8] to the general case.

## 2. Main Results and Example

In this section, we are ready to state and prove the main results.

**Theorem 1 .**

Let {*A*_*n*_}_*n*=1_^+*∞*^ be a periodic sequence with period *p*, and *k*, *r* ∈ {1,2,…} with *gcd*(*k*, *r*) = 1 and *k* ≠ *r*.

1. If *p* ≥ 2 and *k* is odd, then every well-defined solution of (1) is eventually periodic with period *k*.
2. If *p* = 1, then every well-defined solution of (1) is eventually periodic with period *k*.

**Proof**

Let {*x*_*n*_}_*n*=1−*d*_^+*∞*^ be a well-defined solution of (1). It follows from (1) that, for any *n* ≥ 0 and any *i* ≥ 0,

(2) $$\begin{array}{c} x_{(n+1)k+i}=\underset{}{\max}\left\{\frac{A_{(n+1)k+i}}{x_{(n+1)k+i-r}},x_{nk+i}\right\}\geq x_{nk+i}. \end{array}$$

Then, for every *i* ≥ 0, {*x*_*nk*+*i*_}_*n*=0_^+*∞*^ is increasing, and *x*_*nk*+*i*_ < 0 for all *n* ≥ 0 or there exists *N*_*i*_ > 0 such that *x*_*nk*+*i*_ > 0 for all *n* ≥ *N*_*i*_.

We claim that {*x*_*nk*_}_*n*=0_^+*∞*^ is a constant sequence eventually. Indeed, if {*x*_*nk*_}_*n*=0_^+*∞*^ is not constant sequence eventually, then there exist *kr* < *n*_1_ < *n*_2_ < ⋯ such that *x*_*n*_*i*_*k*_ > *x*_(*n*_*i*_−1)*k*_ and *A*_*n*_*i*_*k*_ is a constant sequence for all *i* ≥ 1 since {*A*_*n*_}_*n*=1_^+*∞*^ is a periodic sequence. Thus we have

(3) xni+1k=max⁡⁡{Ani+1kxni+1k−r,x(ni+1−1)k}=Ani+1kxni+1k−r>x(ni+1−1)k≥xnik=max⁡⁡{Anikxnik−r,x(ni−1)k}=Anikxnik−r.

From this we obtain that, for all *i* ≥ 1,

(4) $$\begin{array}{c} \frac{A_{n_{i}k}\left(x_{n_{i+1}k-r}-x_{n_{i}k-r}\right)}{x_{n_{i+1}k-r}x_{n_{i}k-r}}<0. \end{array}$$

It follows that, for all *i* ≥ 1,

(5) $$\begin{array}{c} x_{n_{i}k}x_{n_{i}k-r}=A_{n_{i}k}<0,\;\;\;x_{n_{i+1}k-r}>x_{n_{i}k-r}. \end{array}$$

Therefore we have *x*_*nk*_ *x*_*nk*−*r*_ < 0 eventually. By induction, we can show that *x*_*nk*−*ir*_ *x*_*nk*−(*i*+1)*r*_ < 0 eventually for all 1 ≤ *i* ≤ *k* − 1 and every {*x*_*nk*−*ir*_}_*n*=*r*_^+*∞*^ (1 ≤ *i* ≤ *k* − 1) is not constant sequence eventually.

If *p* ≥ 2 and *k* is odd, then we have *x*_*nk*_ *x*_*nk*−*kr*_∏_*i*=1_^*k*−1^ *x*_*nk*−*ir*_ *x*_*nk*−*ir*_ < 0 eventually. This is a contradiction.

If *p* = 1, then we write *A*_*n*_ = *A* for all *n* ≥ 1 and choose *m*_0_ ≥ *m*_1_ ≥ ⋯≥*m*_2*k*_ such that *x*_*m*_*j*_*k*−*jr*_ < *x*_(*m*_*j*_+1)*k*−*jr*_ and *x*_(*m*_*j*_+1)*k*−(*j*+1)*r*_ = *x*_(*m*_*j*+1_+1)*k*−(*j*+1)*r*_ for any *j* ∈ {0,1,…, 2*k* − 1}. Thus

(6) x(m0+1)k=max⁡⁡{Ax(m0+1)k−r,xm0k}=Ax(m0+1)k−r=Ax(m1+1)k−r=Amax⁡{A/x(m1+1)k−2r,xm1k−r}=x(m1+1)k−2r=x(m2+1)k−2r ⋮=x(m2k+1)k−2kr≤xm0k,

which is a contradiction. This completes the proof of the claim.

By the above claim we may choose an *N* > 0 such that *x*_*nk*_ = *x*_*Nk*_ for all *n* ≥ *N*. Since *A*_*n*_ is a periodic sequence, we can choose an *N*_1_ > *N* such that *A*_*N*_1_*k*+*r*_/*x*_*Nk*_ = max⁡{*A*_*nk*+*r*_/*x*_*Nk*_ : *n* ≥ *N*}. Then, for all *n* ≥ *N*_1_,

(7) $$\begin{array}{c} x_{nk+r}=\max\left\{\frac{A_{nk+r}}{x_{Nk}},x_{(n-1)k+r}\right\}. \end{array}$$

Thus *x*_*nk*+*r*_ = *x*_(*n*−1)*k*+*r*_ for all *n* ≥ *N*_1_ + 1 (otherwise, if *x*_*mk*+*r*_ > *x*_(*m*−1)*k*+*r*_ for some *m* ≥ *N*_1_ + 1, then we have *x*_*mk*+*r*_ = *A*_*mk*+*r*_/*x*_*Nk*_ > *x*_(*m*−1)*k*+*r*_ ≥ *x*_*N*_1_*k*+*r*_ ≥ *A*_*N*_1_*k*+*r*_/*x*_*Nk*_, which is a contradiction). By induction, we can show that *x*_*nk*+*jr*_ is a constant sequence eventually for every 1 ≤ *j* ≤ *k*. Note {*jr* : 1 ≤ *j* ≤ *k*}_mod⁡*k*_ = {0,1, 2,…, *k* − 1} since gcd(*k*, *r*) = 1. Then *x*_*nk*+*i*_ is a constant sequence eventually for every *i* ∈ {0,1,…, *k* − 1}, which implies that {*x*_*n*_}_*n*=1−*d*_^+*∞*^ is eventually periodic with period *k*.

From the proof of Theorem 1 we obtain the following corollary.

**Corollary 2 .**

Let *k*, *r* ∈ {1,2,…} with *k* ≠ *r*. If {*A*_*n*_}_*n*=1_^+*∞*^ is a periodic sequence, then every positive (or negative) solution of (1) is eventually periodic with period *k*.

**Theorem 3 .**

Let {*A*_*n*_}_*n*=1_^+*∞*^ be a periodic sequence with period *p* ≥ 2, and *k*, *r* ∈ {1,2,…} with *gcd*(*k*, *r*) = 1 and *k* ≠ *r*. If *A*_*s*_ ≥ 0 for some *s* ∈ {1,2,…, *p*}, then every well-defined solution of (1) is eventually periodic with period *k*.

**Proof**

Let {*x*_*n*_}_*n*=1−*d*_^+*∞*^ be a well-defined solution of (1). Using arguments similar to the ones developed in the proof of Theorem 1, we know that, for every *i* ≥ 0, {*x*_*nk*+*i*_}_*n*=0_^+*∞*^ is increasing, and *x*_*nk*+*i*_ < 0 for all *n* ≥ 0 or there exists *N*_*i*_ > 0 such that *x*_*nk*+*i*_ > 0 for all *n* ≥ *N*_*i*_.

We may assume without loss of generality that *A*_1_ ≥ 0. We claim that {*x*_*nk*_}_*n*=0_^+*∞*^ is a constant sequence eventually. Indeed, if {*x*_*nk*_}_*n*=0_^+*∞*^ is not constant sequence eventually, then there exist *kr* < *n*_1_ < *n*_2_ < ⋯ such that *x*_*n*_*i*_*k*_ > *x*_(*n*_*i*_−1)*k*_ with *A*_*n*_*i*_*k*_ being a constant sequence for all *i* ≥ 1. Thus we have

(8) xni+1k=max⁡⁡{Ani+1kxni+1k−r,x(ni+1−1)k}=Ani+1kxni+1k−r>x(ni+1−1)k≥xnik=max⁡⁡{Anikxnik−r,x(ni−1)k}=Anikxnik−r,xnpk=max⁡{Anpkxnpk−r,x(np−1)k}≥Anpkxnpk−r.

From this we obtain that, for all *i* ≥ 1,

(9) $$\begin{array}{c} x_{n_{i}k}x_{n_{i}k-r}=A_{n_{i}k},\\ x_{npk}x_{npk-r}\geq A_{1}\geq 0. \end{array}$$

Thus *A*_*n*_*i*_*k*_ ≥ 0 and

(10) $$\begin{array}{c} \frac{A_{n_{i}k}\left(x_{n_{i+1}k-r}-x_{n_{i}k-r}\right)}{x_{n_{i+1}k-r}x_{n_{i}k-r}}<0. \end{array}$$

It follows that, for all *i* ≥ 1,

(11) $$\begin{array}{c} x_{n_{i+1}k-r}<x_{n_{i}k-r}. \end{array}$$

This is a contradiction.

Using arguments similar to the ones developed in the proof of Theorem 1, we can show that *x*_*nk*+*jr*_ is a constant sequence eventually for every 1 ≤ *j* ≤ *k*. Note {*jr* : 1 ≤ *j* ≤ *k*}_mod⁡*k*_ = {0,1, 2,…, *k* − 1} since gcd(*k*, *r*) = 1. Then *x*_*nk*+*i*_ is a constant sequence eventually for every *i* ∈ {0,1,…, *k* − 1}, which implies that {*x*_*n*_}_*n*=1−*d*_^+*∞*^ is eventually periodic with period *k*.

Now we construct an example with *p* ≥ 2 and *k* being even which has a well-defined solution that is not eventually periodic.

**Example 4 .**

Consider the max-type equation

(12) $$\begin{array}{c} x_{n}=\max\left\{\frac{A_{n}}{x_{n-r}},x_{n-k}\right\},\;n=1,2,\ldots , \end{array}$$

where *k*, *r* ∈ {1,2,…} and *k* is even with gcd(*k*, *r*) = 1 and *k* ≠ *r* and *A*_*n*_ is a periodic sequence with *A*_2*i*_ = *A* < *A*_2*i*−1_ = *B* < 0 for all *i* ≥ 1. Choose the initial conditions *x*_−*i*_ = *B* for odd *i* ∈ {0,1,…, *d*} and *x*_−*i*_ = 1 for even *i* ∈ {0,1,…, *d*} with *d* = max⁡{*r*, *k*}; we can obtain a solution {*x*_*n*_}_*n*=−1_^*∞*^ of (12) such that

 (1) If *r* < *k*, then
  (13) $$\begin{array}{c} x_{nk+i}=\{\begin{array}{cc} {\left\{\frac{B}{A}\right\}}^{n}B, & \text{if}\;\;i\in \left\{1,3,\ldots ,r\right\},\\ {\left\{\frac{B}{A}\right\}}^{n+1}B, & \text{if}\;\;i\in \left\{r+2,\ldots ,k-1\right\},\\ {\left\{\frac{A}{B}\right\}}^{n+1}, & \text{if}\;\;i\in \left\{2,\ldots ,k\right\}. \end{array} \end{array}$$

It is easy to verify that lim⁡_*n*→*∞*_ *x*_2*n*_ = *∞* and lim⁡_*n*→*∞*_ *x*_2*n*−1_ = 0.

 (2) If *r* > *k*, then
  (14) $$\begin{array}{c} x_{n2r+i}=\{\begin{array}{cc} {\left\{\frac{B}{A}\right\}}^{n}B, & \text{if}\;\;i\in \left\{1,3,\ldots ,r\right\},\\ {\left\{\frac{B}{A}\right\}}^{n+1}B, & \text{if}\;\;i\in \left\{r+2,\ldots ,2r-1\right\},\\ {\left\{\frac{A}{B}\right\}}^{n+1}, & \text{if}\;\;i\in \left\{2,\ldots ,2r\right\}. \end{array} \end{array}$$

It is easy to verify that lim⁡_*n*→*∞*_ *x*_2*n*_ = *∞* and lim⁡_*i*→*∞*_ *x*_2*n*−1_ = 0.

**Remark 5 .**

Consider the max-type equation

(15) $$\begin{array}{c} x_{n}=\underset{}{\max}\left\{\frac{A_{n}}{x_{n-sr}},x_{n-sk}\right\},\;n=1,2,\ldots , \end{array}$$

where {*A*_*n*_}_*n*=1_^+*∞*^ is a periodic sequence with period *ps* and *s*, *k*, *r* ∈ {1,2,…} with gcd(*k*, *r*) = 1 and *k* ≠ *r*, and *p* = 1 (or *p* ≥ 2), and the initial conditions *x*_1−*d*_, *x*_2−*d*_,…, *x*_0_ are real numbers with $d=\underset{}{\max}\left\{sr,sk\right\}$. Write *y*_*n*_^*i*^ = *x*_*ns*+*i*_ for every 1 ≤ *i* ≤ *s* and *n* = 0,1, 2,…. Then (12) reduces to the equation

(16) $$\begin{array}{c} y_{n}^{i}=\underset{}{\max}\left\{\frac{A_{ns+i}}{y_{n-r}^{i}},y_{n-k}^{i}\right\},\;1\leq i\leq s,\;\;n=0,1,2,\ldots . \end{array}$$

(1) If *p* = 1 (or *p* ≥ 2 and *k* is odd), then it follows from Theorem 1 that, for every 1 ≤ *i* ≤ *s*, every well-defined solution of equation $y_{n}^{i}=\underset{}{\max}\left\{A_{ns+i}/y_{n-r}^{i},y_{n-k}^{i}\right\}$ is eventually periodic with period *k*. Thus every well-defined solution of (15) is eventually periodic with period *sk*.
(2) If *p* ≥ 2 and, for every 1 ≤ *i* ≤ *s*, there exists some *j*_*i*_ such that *A*_*j*_*i*_*s*+*i*_ ≥ 0, then it follows from Theorem 3 that for every 1 ≤ *i* ≤ *s*, every well-defined solution of equation $y_{n}^{i}=\underset{}{\max}\left\{A_{ns+i}/y_{n-r}^{i},y_{n-k}^{i}\right\}$ is eventually periodic with period *k*. Thus every well-defined solution of (15) is eventually periodic with period *sk*.
(3) If *p* ≥ 2 and *k* is even, then it follows from Example 4 that, for every 1 ≤ *i* ≤ *s*, we can construct an equation $y_{n}^{i}=\underset{}{\max}\left\{A_{ns+i}/y_{n-r}^{i},y_{n-k}^{i}\right\}$ such that it has a well-defined solution which is not eventually periodic. Thus we can construct an equation
  (17) $$\begin{array}{c} x_{n}=\underset{}{\max}\left\{\frac{A_{n}}{x_{n-sr}},x_{n-sk}\right\},\;n=1,2,\ldots \end{array}$$

such that it has a well-defined solution which is not eventually periodic.
